# Surgery for necrotizing acute pancreatitis: surgical approach, morbidity and challenges encountered: experience from a tertiary care hepatopancreatobiliary unit in Sri Lanka

**DOI:** 10.3389/fsurg.2026.1709496

**Published:** 2026-02-26

**Authors:** Duminda Subasinghe, Ravindri Jayasinghe, Nilesh Fernandopulle, Vihara Dassanayake, Sivasuriya Sivaganesh

**Affiliations:** 1 Division of HPB Surgery, Department of Surgery, Faculty of Medicine, University of Colombo, Colombo, Sri Lanka; 2 Department of Anaesthesiology and Critical care, Faculty of Medicine, University of Colombo, Colombo, Sri Lanka

**Keywords:** infected pancreatic necrosis, necrotizing acute pancreatitis, pancreatic necrosectomy, pancreatic surgery, resource-limited setting

## Abstract

**Background:**

Infected pancreatic necrosis (IPN) remains a life-threatening complication of acute pancreatitis. While minimally invasive “step-up” strategies are now standard, their implementation in resource-limited settings is often constrained by availability of interventional radiology, advanced endoscopy, and intensive care support. This study describes management pathways, morbidity, and outcomes of surgically treated IPN in a tertiary hepatopancreatobiliary (HPB) unit operating under such limitations.

**Methods:**

A retrospective analysis of prospectively maintained data was performed on patients who underwent surgical necrosectomy for IPN between 2015 and 2021. Management followed a step-up philosophy where feasible, incorporating antibiotics, image-guided or endoscopic drainage, and delayed surgery. Clinical characteristics, interventions, complications, and outcomes were analysed descriptively.

**Results:**

Six patients underwent surgery for IPN. Initial interventions included ultrasound-guided percutaneous drainage (*n* = 3), endoscopic ultrasound-guided drainage (*n* = 1), and primary surgery (*n* = 2). All patients ultimately required open necrosectomy due to persistent sepsis or failure of less invasive measures. Early morbidity was substantial, with organ failure occurring in 83.3%, including acute respiratory distress syndrome in 66.6%. Clinically relevant postoperative pancreatic fistula occurred in 50%, and incisional hernia developed in all patients during follow-up. Median ICU and hospital stays were 17.3 and 58.5 days respectively. There was one mortality (16.6%).

**Conclusion:**

In resource-limited environments, the step-up approach to IPN is frequently constrained by service availability rather than intent. Open necrosectomy remains an essential salvage strategy when minimally invasive interventions are unavailable or unsuccessful, but is associated with significant morbidity. Careful patient selection, delayed intervention, and multidisciplinary management are critical to achieving acceptable outcomes.

## Introduction

Infected pancreatic necrosis (IPN), one of the most severe and life-threatening complications of acute pancreatitis, is characterized by the presence of infected devitalized pancreatic and peripancreatic tissue. Majority (80%) of AP is mild and self-limiting, but in up to 20% it may run a severe course with pancreatic necrosis, responsible for substantial morbidity and mortality rate up to 27% (1). IPN occurs in only 6% of cases of AP but can result in 32%−50% mortality (2). However, 30 % to 70 % of the patients with necrotizing pancreatitis develop infected necrosis (3–5) and eventually, more than 70 % of patients with necrotizing pancreatitis will require a drainage procedure for infected necrosis or persistent symptoms (4).

Despite advances in critical care and surgical techniques, IPN continues to pose significant challenges, with high morbidity and mortality rates. The complexity of managing IPN arises from the need for timely diagnosis, the decision-making involved in timing and selecting appropriate interventions, and the critical balance between surgical and non-surgical approaches.

Conventionally, open necrosectomy has been the mainstay of treatment though it has been associated with considerable morbidity. However, the landscape of IPN management has evolved significantly in recent years with the advent of minimally invasive techniques, including video-assisted retroperitoneal debridement (VARD), and endoscopic transgastric necrosectomy. These aim to reduce surgical trauma and morbidity with resultant better patient outcomes. According to a recent systematic review (5) which included 170 studies comprising 11,807 patients demonstrated lower rate of fistula formation with endoscopic drainage compared to various surgical drainage methods. A lower rate of mortality with endoscopic drainage was also observed in observational studies. The mortality rate was significantly lower in endoscopic drainage compared to open surgery, and compared to minimally invasive access (5). Endoscopic necrosectomy is associated with significantly lower risk of inpatient mortality, adverse events, length of stay, and cost when compared to percutaneous and surgical approaches (5–9). Despite this, open necrosectomy remains a vital tool in the hands of surgeons where access to equipment and expertise in minimal access interventions is limited.

This case series highlights a cohort of patients with infected pancreatic necrosis managed surgically at our institution. The surgical techniques employed, perioperative care, and corresponding outcomes are described. We seek to provide insights into the role of tailored surgical strategies in the management of this complex condition.

## Methods

### Study design and setting

This study was carried out at the HPB surgical unit of a tertiary referral hospital operating within a resource-limited environment. Resource limitations included restricted availability of contrast-enhanced CT due to high demand across emergency and elective radiology services; absence of 24-hour interventional radiology (IR) coverage, particularly during weekends, which limited timely image-guided drainage; and limited ICU bed capacity, which sometimes affected the feasibility and timing of escalation to minimally invasive interventions.

### Patient selection

Patients who underwent pancreatic necrosectomy between 2015 and 2021 for IPN at tertiary care HPB surgical unit in Sri Lanka were included. All patients admitted with acute pancreatitis (AP) from January 2015 to December 2021 were screened. During this period, 56 patients presented with AP, of whom 10 (17.9%) developed radiologically confirmed necrotizing pancreatitis (NP). Among these, 6 patients (60% of NP; 10.7% of all AP) were diagnosed with infected pancreatic necrosis (IPN) based on clinical deterioration, persistent sepsis, or the presence of gas within collections on contrast-enhanced CT, in accordance with the revised Atlanta classification. Management followed a step-up approach whenever feasible. Of the 6 IPN patients, those who improved with antibiotics and supportive care or were suitable for percutaneous or minimally invasive drainage were initially managed non-operatively. Patients who demonstrated persistent sepsis, clinical instability, or failure of minimally invasive interventions were considered for open necrosectomy. All 6 IPN patients ultimately required open surgical necrosectomy and therefore comprised the study cohort.

Medical therapy was pursued as far as possible until clinical status warranted necrosectomy preferably beyond 4 to 6 weeks after the onset of acute pancreatitis. Broad spectrum empirical antibiotic therapy guided by cultures and sensitivity where available, and antifungal therapy were employed. Patients were stepped up from ward to HDU and ICU care as needed and where available.

### Definition and diagnosis of infected pancreatic necrosis

The diagnosis of IPN was established in accordance with the revised Atlanta classification. IPN was diagnosed using one or more of the following criteria:

1. Presence of gas within pancreatic or peripancreatic necrotic collections on contrast-enhanced CT imaging;
2. Positive microbiological culture from fine-needle aspiration (FNA) of necrotic collections, when feasible;
3. Persistent clinical sepsis (fever, leukocytosis, or organ dysfunction) in a patient with radiologically confirmed necrosis when microbiological confirmation was not possible due to resource limitations.

Clinical deterioration was assessed using serial clinical evaluation, inflammatory markers, and imaging findings. IPN was diagnosed based on deteriorating clinical parameters supplemented by inflammatory markers and characteristic appearances on a pancreatic protocol abdominal CT scan.

Surgical access for open necrosectomy was determined by the anatomy of the collection seen on CT and guided by perioperative transabdominal ultrasonography. Where feasible, retroperitoneal access was attempted at initial necrosectomy. Necrotic tissue was removed by a combination of blunt gauze debridement and warm saline irrigation. Pus and necrotic material was sent for microbiological analysis. Drainage was employed using wide-bore (28-32F) chest tubes centred in the necrotic cavity. Slow saline irrigation was continued through the tubes until a clear effluent was obtained. Drains were removed and replaced with stoma bags with clinical improvement, a clear minimal effluent and reduction in inflammatory markers. Where a laparotomy was performed a feeding jejunostomy was also placed.

Post-operatively patients were managed in ICU and stepped down to HDU or wards with clinical improvement. Supplementary parenteral nutrition was administered where necessary and enteral feeding was established at the earliest opportunity using the feeding jejunostomy.

Early postoperative complications were defined as complications occurring within 30 days of surgery or during the same hospital admission, whichever was longer. These included postoperative organ failure, ARDS, AKI, postoperative pancreatic fistula (POPF), intra-abdominal collections, surgical site complications, and any events graded using the Clavien–Dindo classification.

Late complications were defined as complications occurring after 30 days postoperatively or after discharge from the index admission. These included incisional hernia, delayed pancreatic fluid collections, endocrine or exocrine insufficiency, and enterocutaneous fistula formation.

Ethical approval for this study was obtained from the Ethics Review Committee, The National Hospital of Sri Lanka. In-hospital details obtained from patient records (BHTs) were prospectively recorded in an HPB patient database. Intermediate outcomes were obtained during follow-up at the outpatient clinic after discharge. Organ failure associated with acute pancreatitis was defined using the Modified Marshal score (10). Morbidity was classified according to the Clavien Dindo classification (11).

Exocrine failure was diagnosed in the presence of steatorrhoea, watery stools and weight loss which responded to pancreatic enzyme replacement. Long-term follow up was done at the outpatient clinic after discharge. No statistical inference was performed due to the small sample size.

## Results

Six patients (male:female 4:2) underwent surgery for infected pancreatic necrosis. The mean age was 39 years (range 27–44). Although all patients were classified as ASA I–II at the time of surgery, three patients (50%) had significant underlying comorbidities, including diabetes mellitus and Child–Pugh B cirrhosis.

Initial intervention consisted of ultrasound-guided percutaneous drainage in three patients, endoscopic ultrasound-guided drainage in one patient, and primary surgery in two patients due to anatomical constraints or clinical instability. Despite these measures, all patients ultimately required open necrosectomy due to persistent sepsis or failure of less invasive interventions (Table 1).

**Table 1:** Clinico-demographic characteristics and procedure related data.

Patient	1	2	3	4	5	6
Age	28	44	43	44	27	48
Gender	Female	Male	Female	Male	Male	Male
ASA	II	I	II	II	I	I
Aetiology	Idiopathic	Post ERCP	Idiopathic	Alcohol	Alcohol	Gallstones
Timing of initial necrosectomy	8 weeks	6 weeks	7 weeks	6 weeks	5 weeks	8 weeks
Primary access approach	EUS guided drainage (6th week)	USS guided drainage (3rd week)	USS guided drainage (5th week)	Laparotomy	Laparotomy	USS guided drainage (6th week)
Subsequent approach	R/flank (7th week) laparotomy (9th week)	R/flank (6th week) laparotomy (9th week)	R/flank and laparotomy (7th week)	–	L/loin (7th week) ERCP + PD sphincterotomy (3rd month)	L/flank open drainage (8th week) laparotomy (9th week)
Duration of irrigation	15 days	18 days	Not available	10 days	7 days	9 days
Feeding jejunostomy	Yes	No	Yes	No	Yes	Yes
Stoma	Yes	No	No	Yes	No	No
Cultured organism	Coliform	Coliform, Acinetobacter	Coliform	Coliform	Coliform	Coliform

All six patients underwent open necrosectomy via a midline (*n* = 5) or rooftop incision (*n* = 1), combined with a right or left loin incision for retroperitoneal access. Four patients required feeding jejunostomy, and two underwent defunctioning ileostomy following intraoperative colonic injury (Table 2).

**Table 2:** Hospital stay and complications.

Case	1	2	3	4	5	6
Hospital stay (days)	89	59	72	40	55	36
ICU stay (days)	10	5	30	5	36	18
Morbidity Early - Clavien Dindo	Grade III b	Grade III b	Grade IVb	Grade IVa	Grade IVb	Grade V
POPF	None	Yes	Yes	None	Yes	Yes
Organ dysfunction/failure	None	None	ARDS, AKI	ARDS	ARDS, AKI	ARDS, AKI
Incisional hernia	Yes	Yes	Yes	Yes	Yes	Yes

Early postoperative morbidity was substantial. Organ dysfunction occurred in five patients (83.3%), with acute respiratory distress syndrome observed in four (66.6%) and acute kidney injury in three (50%). Clinically relevant postoperative pancreatic fistula developed in three patients (50%). One patient died during the index admission. Median ICU stay was 17.3 days (range 5–36), and median hospital stay was 58.5 days (range 36–89). Clavien Dindo grading of post-operative complications and early and long term morbidity is shown in Table 2.

Late morbidity was dominated by incisional hernia, which developed in all patients during follow-up. The follow-up duration ranged from 18 to 60 months, with a median follow-up of 32 months among the six patients. Endocrine insufficiency occurred in two patients, while no patient developed clinically significant exocrine insufficiency.

Management pathways and outcomes are summarised to demonstrate escalation patterns, perioperative morbidity, and resource-driven decision-making rather than technique comparison.

## Discussion

This case series describes real-world management pathways and outcomes of infected pancreatic necrosis in a tertiary HPB unit operating under resource limitations. Although minimally invasive and endoscopic techniques have become the preferred approach for IPN, their consistent application remains challenging in many low- and middle-income settings. Necrotizing pancreatitis remains a significant local complication of acute pancreatitis. In this cohort, the common etiologies included alcohol (*n* = 2, 33.3%), idiopathic causes (*n* = 2, 33.3%), post-ERCP, and gallstones (16.6% each) (Table 1). Managing necrotizing pancreatitis is inherently challenging due to high morbidity and mortality, which vary depending on baseline comorbidities, the extent and location of necrosis, and the occurrence of complications such as superinfection through gut translocation and multiorgan failure (12).

Although traditional open necrosectomy was long considered the standard therapeutic approach, minimally invasive techniques have increasingly gained prominence due to reports of improved perioperative outcomes (13). The choice of intervention depends on disease severity, anatomic considerations, patient stability, and the expertise and technology available. Regardless of the modality, management goals remain similar but should be individualized. Key priorities include attenuating the systemic inflammatory response, preventing progression to multiorgan failure, and identifying complications related both to the disease and to treatment—such as pancreatic fistulae, hernias, and pancreatic endocrine or exocrine insufficiency—which may lead to prolonged morbidity and repeated interventions (14, 15). Optimal patient care typically requires a multidisciplinary team including interventional radiologists, endoscopists, surgeons, and intensivists (16).

While a step-up philosophy was adopted, implementation was frequently constrained by limited access to 24-hour interventional radiology, restricted availability of advanced endoscopic necrosectomy, and ICU bed shortages. These factors influenced both the timing and feasibility of minimally invasive escalation, resulting in earlier or unavoidable progression to open necrosectomy in selected patients. Consequently, this cohort represents a surgically refractory population rather than the full spectrum of IPN.

Open necrosectomy is now primarily reserved for specific indications such as hemorrhage, abdominal compartment syndrome, hollow viscus perforation, or failure of less invasive modalities, including interventional embolization (15). Early debridement has been abandoned because of high mortality exceeding 50% and a frequent need for reintervention (17). Operative decision-making is influenced by factors such as the presence of infection, clinical stability, extent of necrosis extending into the retroperitoneum, vascular complications, gastrointestinal or biliary obstruction, and pancreatic ascites (18, 19). Minimally invasive techniques—including laparoscopic transperitoneal necrosectomy, video-assisted retroperitoneal debridement (VRAD), natural orifice transluminal endoscopic surgery (NOTES), and percutaneous image-guided drainage—have become increasingly popular (18, 19). These approaches form part of the standard “step-up” algorithm, beginning with percutaneous catheter drainage and escalating to minimally invasive necrosectomy if needed (13, 20). Endoscopic step-up approaches have also demonstrated reduced morbidity in several studies, largely by avoiding surgical trauma, reducing physiological stress, and eliminating the need for general anesthesia (7, 8). The reduced fistula rate associated with endoscopic techniques compared with percutaneous drainage is attributed to the lack of transcutaneous enzyme leakage (9). Persistent organ failure remains a major contributor to poor outcomes in infected pancreatic necrosis, and the physiological insult of open surgery may exacerbate SIRS and multiorgan dysfunction (21).

The PANTER trial, a randomized multicenter study, challenged the role of routine open necrosectomy, reporting lower major morbidity—including perforation, new-onset organ failure, fistulae, and bleeding—in patients treated with a minimally invasive step-up approach as compared to laparotomy, although mortality did not differ between groups (22). Similar multicenter trials support these findings of reduced morbidity without differences in overall survival (8). More recent evidence, however, suggests that outcomes following modern open necrosectomy may be more favorable than earlier reports indicated. In many comparative studies, open surgery was reserved for the most critically ill patients or for those in whom minimally invasive modalities had failed, creating a selection bias that likely contributed to poorer outcomes in the open surgery group (23, 24). With advances in perioperative care, mortality associated with open necrosectomy has decreased (13, 23, 25, 26).

Consistent with contemporary practice, patients in this study initially underwent minimally invasive or endoscopic interventions: one patient (16.7%) had endoscopic drainage (NAGI stent) and five (80%) underwent ultrasound-guided percutaneous drainage. One patient (Case 6) had a ruptured acute necrotic collection with pancreatic ascites, precluding EUS-guided intervention. Despite these measures, all six patients ultimately required open necrosectomy (Table 2). Comorbidity burden was notable, with three patients (50%) having significant underlying disease such as diabetes and Child B cirrhosis (Table 1). Interventional radiologic drainage was used adjunctively in most patients (4/6; 66.6%), and repeated procedures were common (5/6; 83.3%), with a median of two interventions (range 1–4) (Table 2). Morbidity was dominated by organ failure (66.6%), ARDS (83.3%), AKI (50%), and combined ARDS/AKI (50%) (Table 2), findings that are in keeping with published reports from the region, where rates of multiorgan failure up to 53.3% have been described (20). Late postoperative morbidity included incisional hernia (100%) and postoperative pancreatic fistula (50%), with three patients developing Grade B POPF. Delayed pancreatic collections, enterocutaneous fistulae, and endocrine insufficiency occurred in 50% of patients, while no patients developed exocrine failure (Table 3). The incidence of POPF and incisional hernia in this series appears higher than in some regional and randomized studies where reported rates are below 50%; however, given the small sample size and potential selection bias, these findings should be interpreted descriptively. The median hospital stay (58.5 days, range 36–89) and postoperative ICU stay (17.3 days, range 5–30) appear comparable to durations described in other series (5, 20, 21).

Infected necrosis remains an established indication for intervention in necrotizing pancreatitis, with surgical management associated with reduced morbidity and mortality in appropriate cases (27). Up to 40% of patients presumed to have sterile necrosis have been shown to harbor infection on further evaluation (28). In the present series, only three patients (50%) had positive blood cultures, yet all six demonstrated infected necrosis at surgery, consistent with earlier observations (28). The mortality rate in this cohort was 16.6%, which appears comparable to or slightly lower than mortality rates above 20% reported in studies from the region (26) and >10% in randomized trials from Western centers (13, 29). Given the small cohort size and retrospective nature of this study, these observations should be interpreted cautiously and not as formal comparative inferences. Despite this, overall mortality was comparable to regional and international reports, suggesting that acceptable outcomes can be achieved with delayed surgery, meticulous perioperative care, and multidisciplinary management.

This study has several limitations, including its retrospective design, small sample size, and potential selection bias, as not all patients underwent all minimally invasive or endoscopic interventions. Additionally, data were collected over more than five years, during which advances in technology and availability may have influenced management options and diagnostic capabilities.

## Conclusion

Despite increasing global adoption of minimally invasive and endoscopic techniques for infected pancreatic necrosis, their consistent application in low-resource settings remains challenging. In this series, although a step-up strategy was attempted wherever feasible, limitations in interventional radiology, endoscopic availability, and critical care capacity necessitated escalation to open necrosectomy in all patients. Open surgery was associated with substantial morbidity, prolonged ICU and hospital stay, and a high burden of late complications, reflecting both disease severity and selection bias toward surgically refractory cases. Nevertheless, mortality was acceptable and comparable to regional data. These findings reinforce that open necrosectomy continues to play a vital role as a definitive treatment option in selected patients with IPN when minimally invasive approaches fail or are not feasible, particularly in resource-constrained HPB units.

## Data Availability

The original contributions presented in the study are included in the article/Supplementary Material, further inquiries can be directed to the corresponding author.
